# A surprising foreign body abscess

**DOI:** 10.1016/j.idcr.2021.e01108

**Published:** 2021-03-31

**Authors:** Benedikt Kolbrink, Felix Braun, Kevin Schulte

**Affiliations:** a Department of Nephrology and Hypertension, University Hospital Schleswig-Holstein, Arnold-Heller-Str. 3, 24105 Kiel, Schleswig-Holstein, Germany; b Department of General, Thoracic and Transplantation Surgery, University Hospital Schleswig-Holstein, Arnold-Heller-Str. 3, 24105 Kiel, Germany

**Keywords:** Abscess, Nephrectomy, Kidney transplantation, General surgery, Nephrology

## Abstract

An abscess is a common complication after surgical interventions. Furthermore, abscesses of different genesis often occur under immunosuppression. In the case of a kidney transplant patient described here, however, the cause of an abscess-like mass in the abdomen was an unusual one, the etiology of which we could only clarify after surgical removal.

A 38-year-old patient was referred to our transplantation center in northern Germany by his primary care physician, who had sonographically discovered an unclear cystic mass in the left renal fossa. The patient had undergone left-sided nephrectomy in Iraq in 2011 due to uncontrollable nephrolithiasis and had also received a kidney transplant in 2011 due to kidney failure of unclear etiology. Apart from arterial hypertension, his medical history was otherwise unremarkable and he did not report any complaints regarding the mass. He was under immunosuppressive maintenance therapy with prednisolone, cyclosporine and mycophenolate at the time of presentation. CT and MRI scans (Fig. 1) were performed. These showed a 9 × 9 × 8 cm large, encapsulated mass of the former left renal fossa with only very delicate marginal calcification and an atypical inner structure; possible differential diagnoses included echinococcosis, a chronic (amoebic) abscess or an underlying malignancy. However, no elevated infection parameters were found and the serological tests for echinococcosis and amoebiasis were also negative.

**Fig. 1:**
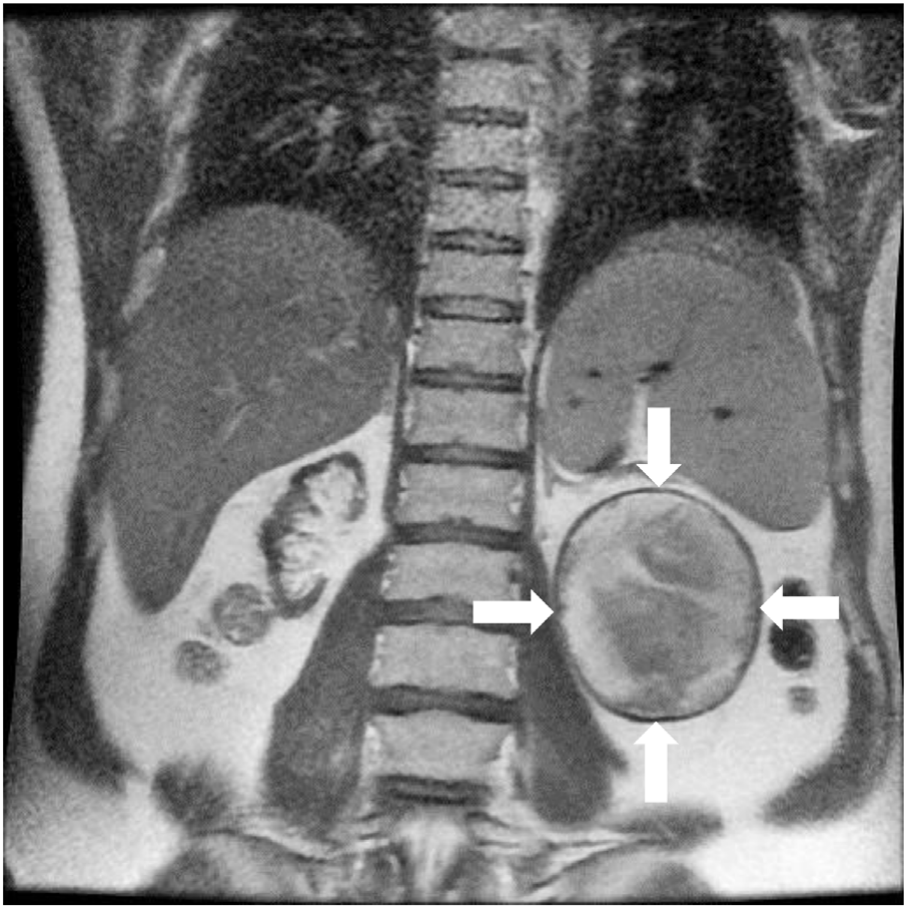
Image of the initially unclear cystic mass in the MRI scan of the abdomen.

After an interdisciplinary team had discussed the further procedure - including a possible referral to the National Reference Center for Tropical Diseases - we decided to surgically remove the mass. Intraoperatively, a coarse, fibrosed tumorous mass presented. After ex situ incision, a significant amount of purulence was evacuated, but even at this time it was still unclear as to what this actually was. No bacteria were detected in the microbiological cultures obtained. Finally, the pathologist's workup of the specimen brought clarity: The cause were gauze swabs (Fig. 2) that had probably been forgotten in the left renal fossa during nephrectomy in Iraq and had now developed over 9 years into this very uncommon tumor. The patient recovered after the operation without any sequelae.

**Fig. 2:**
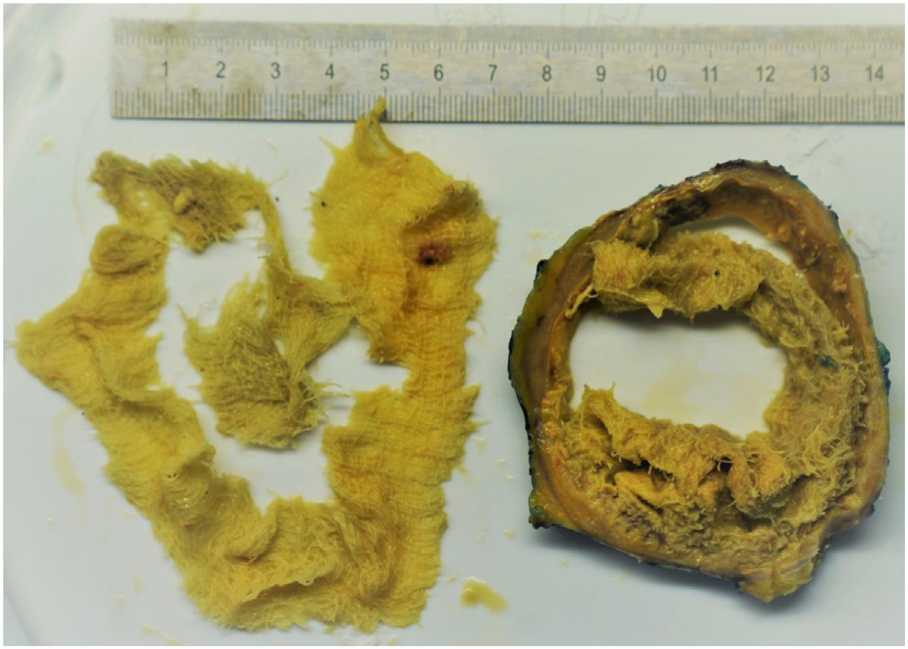
Pathological specimen showing the gauze swabs enclosed by a fibrous capsule.

## Author statement

**Benedikt Kolbrink**: Conceptualization, Writing- Original draft preparation, Visualization; **Felix Braun**: Writing- Review and Editing, Resources; **Kevin Schulte**: Conceptualization, Writing- Review and Editing, Supervision

## Declaration of Competing Interest

None.

